# An Integrated Network, RNA Sequencing, and Experiment Pharmacology Approach Reveals the Active Component, Potential Target, and Mechanism of *Gelsemium elegans* in the Treatment of Colorectal Cancer

**DOI:** 10.3389/fonc.2020.616628

**Published:** 2020-12-23

**Authors:** Lin Wang, Hai-li Xu, Jing-wei Liang, Ying-ying Ding, Fan-hao Meng

**Affiliations:** School of Pharmacy, China Medical University, Liaoning, China

**Keywords:** *Gelsemium elegans*, koumine, PDK1, Akt/mTOR/HK2, glycolysis

## Abstract

In this study, a combination of network pharmacology, bioinformatics analysis, molecular docking and transcriptomics was used to investigate the active ingredient and potential target of *Gelsemium elegans* in the treatment of colorectal cancer. Koumine was screened as the active component by targeting PDK1 through network pharmacology and reverse docking. RNA-Seq, enrichment analysis and validation experiment were then further employed to reveal koumine might function in inhibiting Akt/mTOR/HK2 pathway to regulate cell glycolysis and detachment of HK2 from mitochondria and VDAC-1 to activate cell apoptosis both *in vitro* and *in vivo*. In the present study, we provide a systematical approach for the identification of effective ingredient and potential target of herbal medicine. Our results have important implication for the intensive study of koumine as novel anticancer agents for colorectal cancer and could be supportive in its further structural modification.

## Introduction

Herbal medicine shows promising potential in clinic and plays an important role in disease prevention and treatment (1, 2). Various beneficial effects of herbal medicine have been reported for cancer prevention and treatment, such as extract of the aerial part of Scutellaria barbata BZL101 for patients with advanced breast cancer (3) and PHY906 (a pharmaceutical-grade formulation of four traditional Chinese herbs) in the treatment of advanced hepatocellular carcinoma (4). However, it is rather difficult to clarify the pharmacological effect of herbal medicine because it contains multiple chemical constituents. Therefore, the search for a targeted, effective ingredient is an urgent need.

*Gelsemium elegans* (Gardner&Champ.) Benth. (*G. elegans*) belonging to the Gelsemiaceae family is an extremely poisonous plant (5). It has attracted more attention for various therapeutic effects on anti-inflammatory, analgesic, anxiolytic and immunostimulatory activity (6–10). In recent years, a rich body of evidence has also demonstrated that *G. elegans* possesses anti-tumor effects *in vivo* and *in vitro* (11–13). Remarkably, it has also been applied in the treatment of colorectal cancer (CRC) in the folk for hundred years. Nevertheless, *G. elegans* has been greatly limited because of its toxicity and narrow therapeutic window. So far, almost 200 compounds have been separated from *G. elegans*, including alkaloids, iridoids, triterpenes, phenolic acids, steroids, coumarins, lignans, megastigmane glycosides, and other ingredients (14). Despite the extensive and rapid progress in active ingredient research, there is still an ever-growing need for efficient strategies to identify the effective ingredient and potential target of *G. elegans* for CRC treatment.

In the present study, an integrative pharmacology method including network, molecular docking, transcriptomics, bioinformatics analysis and immunoblot analysis was used to reveal the active component, potential target and mechanism of *G. elegans* on CRC. As a result, koumine (KM) which is the most abundant molecule among the alkaloids of *G. elegans* with relatively low toxicity was identified the active agent by targeting 3-phosphoinositide-dependent protein kinase-1 (PDK1). It inhibited glycolysis and interaction of hexokinase 2 (HK2) with voltage-dependent anion channel-1 (VDAC-1) on the mitochondria *via* suppressing its downstream Akt/mTOR/HK2 pathway. KM exhibited good lead compound properties and provide a great potential scaffold for future structure modification in developing highly efficient anti-CRC agents.

## Method

### Network Construction and Enrichment Analysis

**Figure 1:**
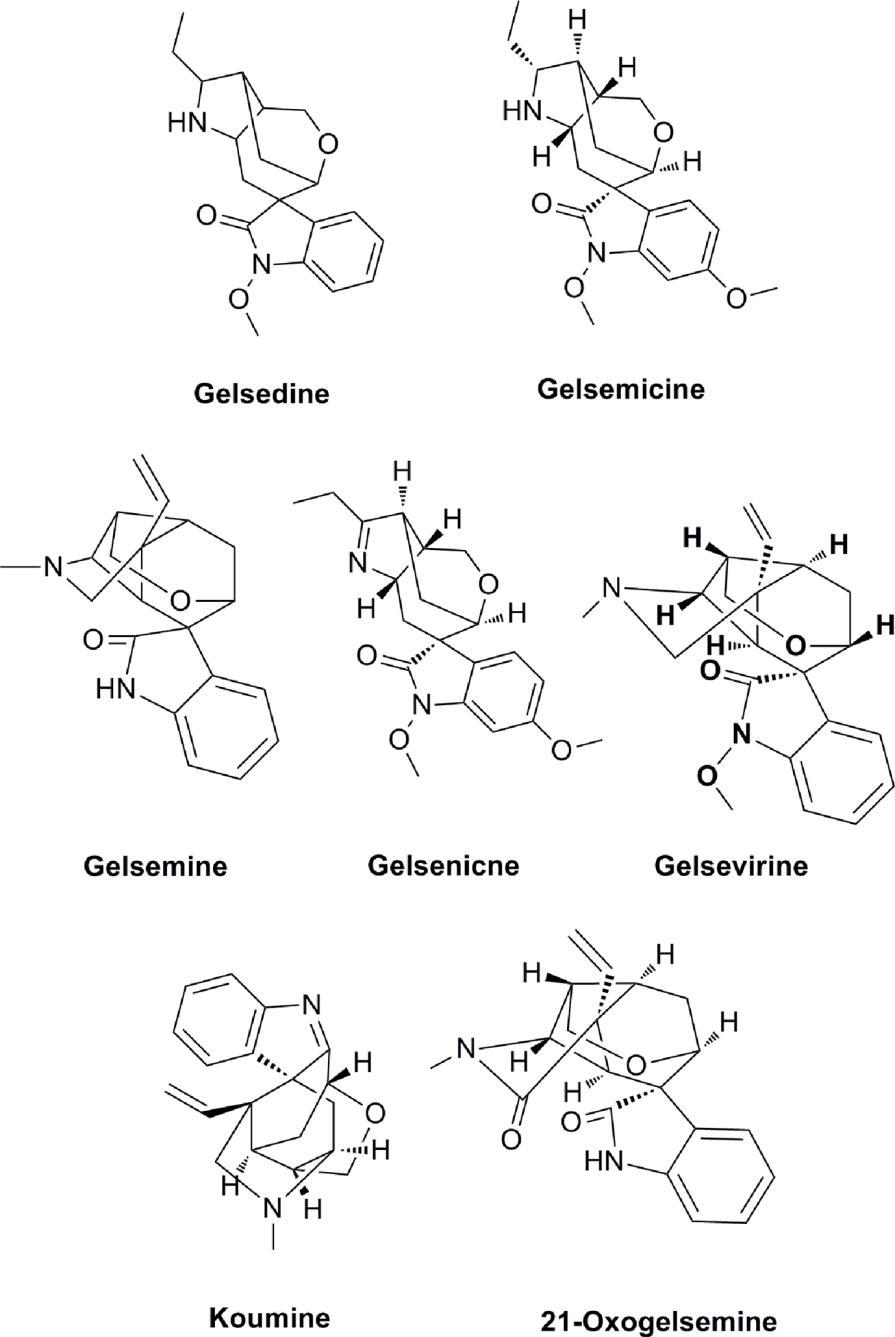
Seven effective components of *G. elegans*.

The network construction and enrichment analysis was conducted according to our previous research method with minor improvements (15). A total of seven compounds (**Figure 1**) with significant pharmacological activities and high contents from *G. elegans* were selected. We imported the components’ chemical structure into the public network server of the database PharmMapper (http://www.lilab-ecust.cn/pharmmapper/) which employs pharmacophore mapping strategy for the identification of the potential targets. The top 50 targets of each compound were selected for further study of the comprehensive network pharmacology analysis. The compound targets were imported into UniProt knowledgebase (https://www.uniprot.org/uploadlists/) to obtain official symbols for the following enrichment analysis.

The online database STRING (https://string-db.org/) was applied to construct the protein–protein interactions (PPI) analysis by importing the potent targets. The minimum required interaction score was set higher than 0.4. KEGG and GO enrichment analysis were carried out to identify the candidate targets. The cut-off criterion was set with the *P* values less than or equal 0.05 and the result of GO enrichment was only showed by the Biological Process terms.

### Molecular Docking

The molecular docking study was conducted on Molecular Operating Environment package (MOE 2015.10). The structure of compounds was drawn and converted into 3D structure by ChemBio 3D Ultra 14.0 and the candidate targets crystal structure was extracted from the Protein Data Bank (http://www.rcsb.org/pdb/home/home.do). The ligand conformations were generated with the bond rotation method. The placement and refinement were set as “Triangle matcher” and “Induced Fit” respectively. The best docking conformation was selected from 30 predicted poses with the best binding affinity according to their E-strain and the London dG score was applied to evaluate ligand binding capacity to the receptor.

### Cell Culture

The human CRC cell lines including HCT116 and HT29 were obtained from American Type Culture Collection (ATCC, Mansassas, VA, USA). All cells were grown in culture utilizing RPMI-1640 medium supplemented with 10% fetal bovine serum (Gibico; Thermo Fisher Scientific, Inc.), 100 μg/ml streptomycin and 100 U/ml penicillin (Sigma Aldrich, St. Louis, MO, USA). Cells were maintained in a humidified atmosphere with 5% CO_2_ at 37°C.

### MTT Assay

(4,5-dimethylthiazol-2-yl)-2,5-diphenyltetrazolium bromide (MTT) assay was carried out to evaluate the viability of HCT116 and HT29 cells. Cells in log phase was seeded in a 96-well plate at a density of 3 × 10^3^ cells/well in 100 μl of complete culture medium and incubated in the presence of KM with increasing concentrations (0, 5, 10, 25, 50, 75, 100, 125 μM) at 37°C for 24 h, respectively. After incubation, MTT solution (10 μl of 5 mg/ml) (Boster Biotechnology Limited Company, Wuhan, China) was added to each well and maintained at 37°C for additional 4 h. Subsequently, the supernatant was removed and 100 μl dimethylsulfoxide (DMSO) was added to dissolve the formazan crystal. The optical density (OD) at 490 nm was measured on microplate reader (Bio-Rad Laboratories, Inc., Hercules, CA, USA).

### Flow Cytometry Analysis of Apoptosis

HCT116 and HT29 cells were seeded in six-well plates overnight, and then treated with different concentration of KM (0, 25, 75, and 125 μM) for 24 h. The cells were harvested, washed twice with ice-cold PBS, and evaluated for apoptosis by double staining with 5 μl Annexin-V FITC (Promega Corporation, Madison, WI, USA) and 5 μl propidium iodide (Promega Corporation, Madison, WI, USA) in 500 μl binding buffer for 15 min in the dark. Stained cells were analyzed by flow cytometer (BD Biosciences, CA, USA).

### PDK1 Kinase Assay

Different concentration of KM in 50 μl kinase reaction buffer (50 mM HEPES, pH 7.5, 10 mM MgCl_2_, 0.1 mg/ml BSA, 2 mM DTT, 1% DMSO) were incubated with 5 ng/ml human recombinant PDK1 kinase (Invitrogen Corporation, Carlsbad, USA). The reaction was subsequently initiated by the addition of 5 μM ATP and 50 μM PDKtide substrate with the sequence: KTFCG TPEYL APEVR REPRI LSEEE QEMFR DFDYI ADWC. Kinase-Lumi™ Luminescent Kinase Assay Kit (Beyotime Biotechnology Company, Shanghai, China) was used for the PDK1 inhibitory activity as described in the manufacturer’s protocol. After incubation for 30 min at room temperature, the luminescence value was measured by chemiluminescence module of a full-wavelength multi-function microplate reader (PerkinElmer, Singapore). The amount of luminescence from each reaction is inversely correlated with PDK1 kinase activity.

### RNA Sequencing

After DMSO or KM treatment, HCT116 cells were collected and washed with cold PBS for twice. A total of six samples were available, including three samples of KM group and three sample of control group. Total RNA was extracted by TRIzol reagent following the manufacturer’s instructions (Takara, Dalian, China). Library construction and mRNA sequencing were carried out by Personal Gene Technology Company (Nanjing, China). mRNA was enriched from total RNA by Oligo (dT) beads. The enriched mRNA underwent fragmentation and was reverse transcribed into cDNA. Then the cDNA fragments were purified with AmPure XP system (Beckman coulter, Beverly Hills, California, USA) and poly-A was added and ligated to Illumina sequencing adapters. The ligation products were evaluated using the Agilent 2100 Bioanalyzer (Agilent, Palo Alto, CA, USA) and sequenced on Illumina Hiseq Xten platform.

The differentially expressed genes (DEGs) were calculated by DESeq (version 1.30.0). The *P*-value was adjusted by the false discovery rate (FDR). P-value <0.05 and the fold change **≥**2 were set to be statistically significant. Gene ontology (GO) functional enrichment and Kyoto Encyclopedia of Genes and Genomes (KEGG) pathway analyses were performed for the bioinformatics analysis.

Details of reverse transcription-quantitative polymerase chain reaction (RT-*q*PCR) are given in **Supplementary Materials**.

### Glucose Uptake Level and ATP Production Assay

The cells were incubated in 6-well plates at a density of 70–80% and treated with various concentrations of KM for 24 h. The glucose level in the medium was measured by glucose assay kit (Nanjing Jiancheng Bioengineering Institute, Nanjing, China) according to the manufacturer’s instructions. Then the concentrations of glucose uptake in each sample were calculated. Intracellular ATP level was estimated using an ATP assay kit (Nanjing Jiancheng Bioengineering Institute, Nanjing, China), strictly following the manufacturer’s instructions. The relative glucose consumption rate and ATP production were normalized by the cell counts and protein concentration of samples, respectively. All experiments were performed for three times.

### Western Blotting Analysis

Cells were harvested in RIPA lysis buffer (Thermo, MA, USA) containing protease and phosphatase inhibitors at 4°C. Protein extract was prepared according to the instruction of extraction kit (Beyotime Biotechnology Company, Shanghai, China). BCA protein assay kit (Beyotime Biotechnology Company, Shanghai, China) was used to quantify the protein. Then equal total amounts of proteins were separated by sodium dodecyl sulfate-poly-acrylamide gel electrophoresis and transferred onto the PVDF membrane (Bio-Rad Company, USA). After blocking with 5% non-fat milk, the membranes were incubated with specific primary antibodies against the indicated protein overnight at 4°C, and washed with PBST, followed by HRP-conjugated secondary antibodies for 1 h at room temperature. Protein bands were developed using the enhanced chemiluminescence reagent and scanned by the Bio-Rad Gel Doc 2000 gel imaging system with *β*-actin band as the reference.

### Immunofluorescence Staining

Cells cultured on glass coverslips were washed with PBS, fixed in 4% paraformaldehyde for 30 min and permeabilized with 0.5% Triton X-100 for 20 min. Then cells were washed twice with PBS and blocked with 3% BSA for 30 min at room temperature, followed by incubation with primary antibody overnight at 4°C. After washing three times for 5 min, the cells were stained with FITC/Texas Red-conjugated secondary antibodies (1:200 dilution; Proteintech, Chicago, IL, USA) for 1 h in the dark. The mitochondria of the cells were exposed to Mitotracker Red (Molecular Probes, Inc., Eugene, OR) at a final concentration of 150 nM for 10 min at 37°C prior to fixation in the dark. After washing three times for 5 min, cells were counterstained with DAPI for 5 min in the dark and examined under confocal fluorescence microscopy (C2; Nikon, Tokyo, Japan).

### Tumor Xenograft Model

All the experimentation for animals was complied with the approval and guidelines of the China Medical University Institutional Animal Care and Use Committee. BALB/c nude mice (six-week-old, male) were obtained from Huafukang Bioscience. CO. Inc (Beijing, China). HCT116 cells in 200 μl (5 × 10^7^ cells/ml) were implanted subcutaneously into the left axillary of each nude mice. When the tumor volume reached about 100 mm^3^, the mice were randomly divided into two groups (n = 8) and intraperitoneally injected with vehicle control (saline) or KM (2 mg/kg) every two days for 3 weeks. The body weight and tumor sizes were measured every other day. The tumor volume was determined by measuring length (L) and width (W) and calculated by the formula: tumor volume (mm^3^) =L*W^2^/2. After the experiment, mice were sacrificed and the tumors were weighed and photographed. The freezing tissues were fixed with 4% paraformaldehyde in 4°C in the purpose of preparing for subsequent immunohistochemical studies.

### Immunohistochemistry Assay

After fixation, dehydration, and transparency, the tumor was embedded in paraffin and cut into 5 um thick sections. Tumor tissue sections were dewaxed in xylene, hydrated with gradient alcohol, 3% hydrogen peroxide blocked endogenous peroxidase and then 0.01 M citrate buffer solution (pH6.0) was used for antigen retrieval. Next, the sections were blocked with 3% fetal bovine serum, and incubated the primary antibody at 4°C overnight. After washed and incubated with the biotinylated secondary antibodies for 1 h at 37°C, the sections were added the horseradish peroxidase-labeled streptomycin avidin working solution to incubate at 37°C for 30 min. Then sections were washed with PBS, developed with diaminobenzidine (DAB), counterstained with hematoxylin, and finally imaged with a light microscope (ECLIPSE E600; Nikon, Tokyo, Japan).

### Statistical Analysis

All statistical analysis was performed by SPSS20.0 software. All the presented data and results were confirmed in at least three independent experiments and expressed as mean ± SD. Statistical analysis of data was made by the student’s t-test or one way ANOVA. *P <* 0.05 is considered statistically significant.

## Results

### Acquisition of Potential Active Component and Target of *G. elegans*

**Figure 2:**
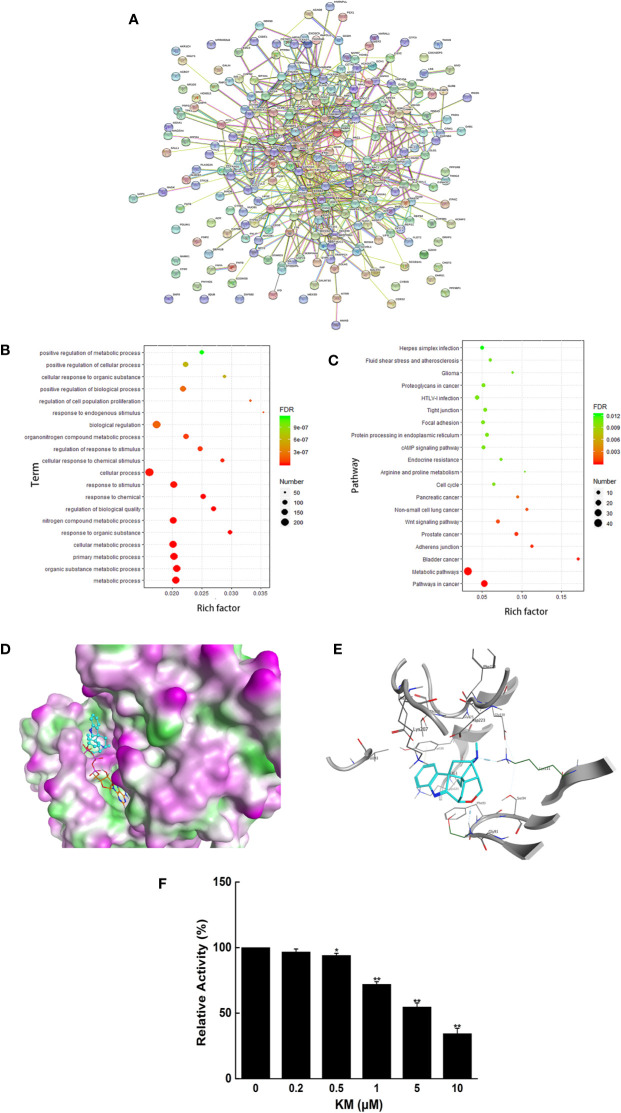
**(A)** Clusters of screened PPI networks **(B)** The 20 representative terms with the lowest *P* value of biological process (BP) gene ontology enrichment. **(C)** The top 20 pathways with *P <*0.05 of KEGG pathway enrichment. **(D)** Molecular interaction of KM with PDK1. **(E)** The 2D representation of KM docking pose with PDK1. **(F)** The effect of KM on PDK1 kinase activity. Data were shown as mean ± SD (n = 3); significance: *P < 0.05, **P < 0.01 *vs* control.

After overlapping the results of seven ingredients from the PharmMapper database, structure of 289 predicted targets were downloaded from the PDB database by employing the Retrieve/ID mapping. As shown in **Figure 2A**, a total of 244 nodes, and 678 edges were mapped in the PPI network of the target genes.

GO analysis was performed to understand the relationship between functional units and their underlying significance in biological system networks. According to the GO enrichment analysis, 561 gene ontology terms were significantly associated with a large number of biological processes (**Supplementary Materials Table 1**), the 20 representative terms with the lowest *P* value in biological process groups was exhibited in **Figure 2B**. The results revealed that the target genes were mainly enriched in the biological process related to the metabolism including metabolic process, organic substance metabolic process and cellular metabolic process. Moreover, through KEGG analysis constructed on *P* value less than 0.05 (**Supplementary Materials Table 2**), top 20 pathways were also obtained (**Figure 2C**), which were associated with metabolic pathways, pathways in cancer and so on. After integrating the results of GO and KEGG analysis, the genes TP53 and PDK1 enriched in the above signaling pathway and closely related to metabolism were picked as candidate targets for further docking research.

The docking results of seven compounds from *G. elegans* were screened out by sorting the docking scores in descending order (**Supplementary Materials Table 3**). KM exhibited adequate spatial orientation in the active site and superior binding capacity to PDK1 with the score −9.1004. It is apparent KM formed hydrogen bond with the backbone of Lys111 and Glu130 located in the kinase domain (**Figures 2D, E**). The kinase activity assay also validated KM possessed the potential inhibitory effect on PDK1 (**Figure 2F**). These results provided great support that KM contributes to the potential anti-CRC effect *via* targeting PDK1.

### KM Induced Apoptosis of CRC Cells

**Figure 3:**
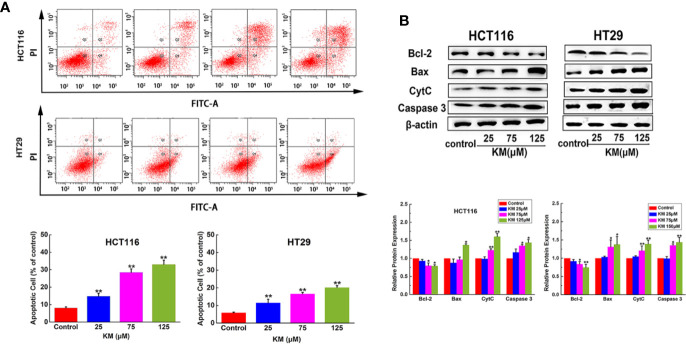
KM promoted CRC cells apoptosis in a dose-dependent manner. **(A)** Flow cytometric analysis of HCT116 and HT29 cell apoptosis after incubation with KM for 24 h. **(B)** Western blot and quantification of proteins associated with apoptosis (Bcl-2, Bax, Cyt-C and caspase 3) in HCT116 and HT29 cells after incubation with KM for 24 h. Data were presented as mean ± SD (n = 3); significance: *P < 0.05, **P < 0.01 *vs* control.

The MTT assay was performed to validate the effect of KM on cell viability. KM treatment exhibited loss of cell viability to HCT116 and HT29 cells with IC_50_ value of 70.56 and 62.82 μM, respectively (**Supplementary Materials Figure 1**). The cells were evaluated by V-FITC and PI double labeling for apoptosis evaluation. Significant increase in the rate of apoptosis was observed from 8.1% (control group) to 33.0% for HCT116 cells after treatment with KM at 125 μM. The effect exhibited a concentration-dependent increase. Similar results were obtained for another CRC cell line HT29 (**Figure 3A**).

In order to confirm this cell death was apoptotic, apoptosis-related proteins were assessed by western blotting with the absence or presence of KM. The results showed the hallmark protein of apoptosis, including Bax, CytC and Caspase-3 expressed a significantly dose-dependent increase. Moreover, Bcl-2, known as a cell survival protein, remarkably decreased depending on the concentration of KM (**Figure 3B**). These results showed that KM activated the apoptotic pathway *via* decreasing Bcl-2 expression associated with an increase in Bax, CytC and Caspase-3.

### KM Suppressed Glycolysis of CRC Cells

**Figure 4:**
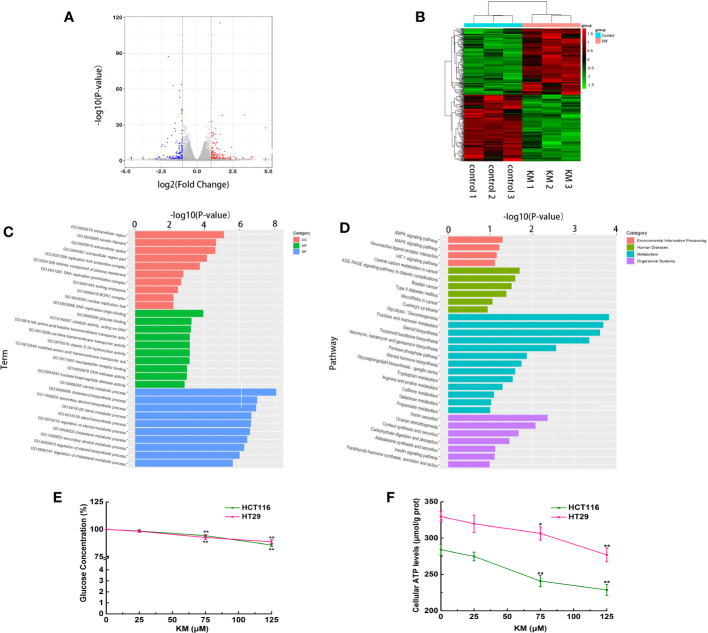
**(A)** Volcano plot of the DEGs with FDR ≤0.05 and the fold change ≥2. Red dots indicate upregulated DEGs; blue ones indicate downregulated DEGs, and gray ones indicate no significant difference. **(B)** Heatmap of expression profile for the 306 DEGs (153 upregulated and 153 downregulated). Each row represents a mRNA and each column represents a sample. **(C)** Gene ontology (GO) enrichment analysis of DEGs. **(D)** Kyoto Encyclopedia of Genes and Genomes (KEGG) pathway enrichment analysis of DEGs. **(E)** Effect of KM on glucose consumption. **(F)** Effect of KM on production of cellular ATP. Data were presented as mean ± SD (n = 3); significance: *P < 0.05, **P < 0.01 *vs* control.

The total gene expression was analyzed by RNA-Seq of HCT116 cells. The genes and pathways changed in response to KM treatment were revealed by this high-throughput method. A total of 306 differential expression genes (DEGs) were identified, including 153 downregulated genes and 153 upregulated genes (using twofold as the cut off value, **Supplementary Materials Tables 4** and **5**). The volcano plot map showed significant genes expressed in the KM treated group compared to the control group (**Figure 4A**). The heatmap used for hierarchical clustering analysis (HCA) exhibited distinct gene expression between control and treated group (**Figure 4B**).

GO functional enrichment was performed to explore the potential molecular or biological functions affected by KM treatment. All the DEGs were categorized into three major categories: cellular components, molecular functions, and biological process. The significantly enriched GO biological processes by the KM treatment included sterol, steroid and cholesterol metabolic and biosynthetic process (**Figure 4C**). Treatment with KM also altered glycometabolism by regulating key genes involved in glucose binding and fructose-bisphosphate aldolase activity like HKDC1, HK2, ALDOA and ALDOB. These results suggested KM might function in regulating the cell energy metabolism. These findings were further confirmed by KEGG pathway analysis. The top 20 pathways of KEGG assignments are listed in **Figure 4D**. At the transcriptomic level, 17 pathways were significantly (*P* < 0.05) enriched for gene expression in the KM treated and control group comparisons which showed that glycolysis/gluconeogenesis, fructose and mannose metabolism and steroid biosynthesis were highly enriched among the DEGs resulting from KM treatment. To confirm the DEGs identified by RNA-Seq, *q*RT-PCR validation was performed on the representative genes in metabolism pathway. The mRNA expressions of these genes were all consistent with the results of RNA-Seq (**Supplementary Table 6** and **Figure 2**).

To further confirm the results of RNA-Seq, glucose concentration in the medium and intracellular ATP level were examined after both HCT116 and HT29 cells were exposed to KM for 24 h. As shown in **Figures 4E, F**, the glucose uptake in KM treated cells was significantly lower than that in untreated control group (*P* < 0.05), meanwhile, the intracellular ATP generation of KM treated group was significantly decreased compared to control group (*P* < 0.05). An integrated analysis of mentioned above revealed that KM may facilitate the CRC cell apoptosis effect *via* suppression of the glycolysis process.

### KM Inactivated the Akt/mTOR/HK2 Pathway and Promotes HK2 Disassociation to VDAC-1 *via* Targeting PDK1

**Figure 5:**
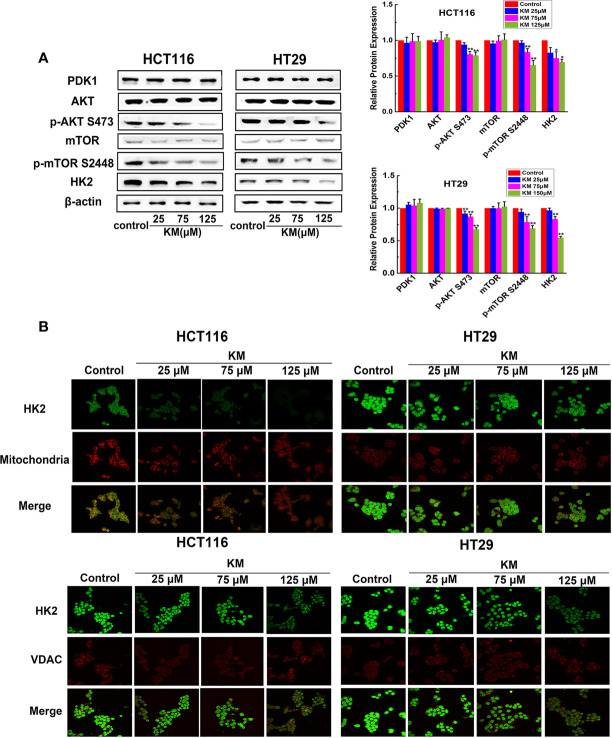
KM repressed glycolysis and promoted the dissociation of HK2 from mitochondria and VDAC by downregulating Akt/mTOR/HK2 pathway in CRC cells. **(A)** Effect of KM on the expression level of Akt/mTOR/HK2 pathway by targeting PDK1. Protein levels were measured by Western blot analysis. **(B)** Representative immunofluorescence confocal images of CRC cells labelled with HK2, VDAC antibody and Mito-Tracker. Data were presented as mean ± SD (n = 3); significance: *P < 0.05, **P < 0.01 *vs* control.

In accordance with the suppression of tumor glycolysis, the expression of HK2 which is the key enzymes of glucose metabolism was markedly decreased in a dose-dependent manner (**Figure 5A**). In order to further understand the potential molecular mechanism by which KM modulating HK2 expression on CRC cell lines, we focused on the Akt/mTOR pathway, which is a widely acknowledged signaling cascade interacting with a wide variety of cellular processes including glucose metabolism of cancer cells and serve as a functional link between PDK1 and HK2. As shown in **Figure 5A**, phosphorylation of Akt and mTOR was dramatically downregulated, while KM had no effect on the expression levels of PDK1, total Akt, and mTOR after KM treatment. Taking these results together, KM might inhibit glycolysis through suppressing Akt/mTOR/HK2 pathway *via* targeting PDK1 in CRC cells.

HK2 is particularly important for the catabolism of glucose as it is the first enzyme of glycolysis (16). Moreover, HK2 is located on the outer membrane of the mitochondria *via* its binding to VDAC-1 to exert it biological function (17). The immunofluorescence was performed to detect the change of both HK2-mitochondria and HK2-VDAC-1 association of CRC cells. Intense green staining of HK2 was detected in the control group while HK2 staining co-localized with red Mitotracker staining in merged images. The HK2 intensities were dramatically decreased in response to KM exposure and a significant attenuation of HK2 associated with mitochondria was observed in merged images. Meanwhile, formation of the HK2 dot-like structures became lower pronounced and less of these structures co-localized to the VDAC-1 foci (**Figure 5B**). The above observations together suggested that the suppression of Akt/mTOR pathway is responsible for the downregulation of HK2 and resulted in the disruption of HK2-VDAC combination which is conducive to metabolic activity and survival.

### KM Inhibited Tumor Growth in an HCT116 Xenograft Mouse Model

**Figure 6:**
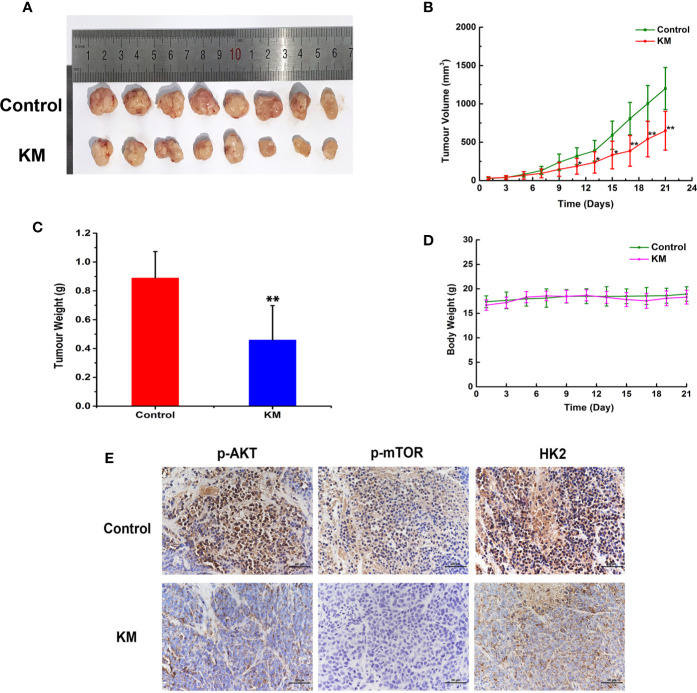
KM inhibited colorectal tumor progression by Akt/mTOR/HK2 pathway in CRC xenograft mouse models. **(A)** Photograph of tumors in vehicle and KM treated group. **(B)** Tumor growth curve of control and KM treated group. **(C)** Tumor weight of control and KM treated group **(D)** The change of body weight of tumor bearing mice. **(E)** Immunohistochemical staining detection of p-Akt, p-mTOR and HK2 in tumor sections from control and KM-treated mice. All panels are of the same magnification. Data were shown as mean ± SD (n = 8). significance: *P < 0.05, **P < 0.01 *vs* control.

To assess the effect of KM against CRC, a subcutaneous xenograft model of HCT116 cells was established in BALB/c nude mice. After 21 days treatment, the average tumor size of the control group was 1,200 ± 237 mm^3^ compared with 650 ± 253 mm^3^ of KM-treated group which resulted in a significant decrease in tumor volume (*P* < 0.05, **Figures 6A, B**). The average tumor weights of KM-treated group were significant smaller than those in the control group (*P* < 0.05, **Figure 6C**). Meanwhile, no obvious toxicity was observed as evaluating the change of body weight (**Figure 6D**). Moreover, immunohistochemistry staining assay showed intensity of staining of p-AKT, p-mTOR and HK2 were substantially lower after KM-treated group (**Figure 6E**) which indicated the expression of them was decreased after KM treatment. Taken together, these results indicated that 2 mg/kg KM showed effective inhibition on the xenograft tumor growth which was in accordance with the mechanisms *in vitro*.

## Discussion

Herbal medicine has long been recognized for their therapeutic properties and serves as an important source for chemical entities supporting drug discovery (18). Even though, the active ingredient and its mechanism have not been extensively investigated that limits their application, especially for the herbal medicine with toxicity. The traditional methods for these studies were based on the separation and random screening with lower reproductive and throughput. It is of great significance and economic value to provide fast, efficient and high throughput approach to acquire the active ingredient and its targets of herbal medicine. The chemical constituents of *G. elegans* have been studied since the early 1930s and it has attracted a great deal of attention on cancer treatment because of their novel structures and diverse biological activities. However, the molecular mechanism of its action on CRC still was obscure. In the present study, we aimed of providing a novel, efficient and systematic strategy to identify the active ingredient and its potential molecular mechanism. Network-based pharmacology coupled with molecular docking and bioinformatics analysis were successfully applied in predicting the active ingredient and its target of *G. elegans* on CRC. As a result, KM, the most abundant molecule among the alkaloids of *G. elegans*, was deemed to be the potential effective compound with targeting PDK1.

PDK1 has been implicated in cancer progression, including proliferation, apoptosis and invasion and involved in signaling pathways altered in cancer, such as PI3K/Akt, Ras/MAPK and Myc (19). Moreover, over-expression of PDK1 was observed at both mRNA and protein levels in CRC specimens and cell lines (20). Thus, targeting PDK1 provides great therapeutic strategy for CRC. PDK1 is a soluble and globular protein composed of 556 amino acid and possesses an N-terminal Ser/Thr kinase domain and a C-terminal pleckstrin homology (PH) domain. The kinase domain contains two crucial elements for the regulation of kinase activity. One is the phosphorylation of serine 241 in the large C-terminal lobe that is catalyzed by PDK1 in *trans* and necessary for its kinase activity. Another is the PIF pocket located at the N-terminal lobe which could bind a hydrophobic motif on many PDK1 substrates (21). KM was a monoterpene indole alkaloid with a cage structure composed of six rings. Notably, KM exhibited the highest distribution in intestine *in vivo* in our previous study (22) which was consistent with the predicted results. Molecular docking result reveals that KM formed a stable complex with PDK1 at the adjacent domain of the ATP catalytic core region. The piperidine nitrogen atom establishes a hydrogen bond interaction with the backbone of Lys111. Lys111 which coordinates the *α* and *β*-phosphate of ATP with hydrogen bond has an ion pair interaction with absolutely conserved Glu130 located within the *α*C-helix (23). The integrity of Lys111 and Glu130 is crucial for the catalytic activity. There is another hydrogen bond interaction between the oxygen atom and Ser92. As a result, we speculated KM had a competitive interaction with ATP in the ATP catalytic site. In addition, it may also bind to the kinase domain in an allosteric pocket adjacent to the ATP-binding site. The specific mechanism remains to be further explored.

RNA-seq mainly focused on transcribed regions of genomes, serves as a powerful tool for exploring differentially expressed genes following treatment with various compounds and evaluating interactions between compounds and biological systems to reveal molecular mechanism (24). Our studies had shown that induction of apoptosis was a key molecular mechanism responsible for the anti-CRC activity of KM. To explore the possible downstream signaling of KM related to PDK1-induced CRC cell apoptosis, both RNA-seq associated with bioinformatics data-mining tools and experimental verification was performed. The DEGs were mainly enriched in the GO analysis corresponded to biological process such as the steroid metabolic and biosynthetic process and KEGG pathways related to metabolism including glycolysis/gluconeogenesis and fructose, mannose metabolism and steroid biosynthesis. The growth of malignancies including CRC prefers to metabolize glucose by glycolysis even in normoxic condition to meet high biogenetic demands in support of rapid and uncontrolled growth (25, 26). Such a reprogrammed cellular metabolism, known as ‘‘Warburg effect’’, is now recognized as a key hallmark of cancer which not only promotes tumor cell growth and spread but also relapse and chemotherapy resistance (27). As a result, targeting tumor glycolysis pathway has the potential to be a scientifically cancer treatment approach (28, 29). It is worth pointing out that KM determined a dramatically dose-dependent glucose uptake and ATP generation reduction. We also observed KM significantly downregulated the mRNA and protein levels of HK2, an enzyme responsible for the first step of glycolysis. These results revealed that KM has the capacity to suppress glycolysis by decreasing HK2 expression. It is generally accepted that HK2 has a dual role in tumor cells that HK2 could not only result in cell growth *via* enhanced glycolysis but also inhibit apoptosis *via* binding of VDAC-1 to the mitochondrial outer membrane. With HK2 dissociating from VDAC-1, the integrity and permeability of mitochondria membrane was disrupted which activated a release of pro-apoptotic enzyme, such as cytochrome C. It was well documented in our study that exposure to KM resulted in the decrease of the interaction between HK2 and VDAC-1, which could induce CRC cells to undergo apoptosis.

Furthermore, we investigated the pathway of KM involved in the inhibition of HK2 by targeting PDK1. Protein kinase B (Akt), one of the most defined PDK1 targets relevant in human cancer, is involved in the regulation of cellular survival through promoting glucose metabolism by stimulating the activity of hexokinase (30). Of note, studies have documented Akt/mTOR pathway has been a metabolic regulator center of cancer that could impede glycolysis and restrain cancer progression by modulating HK2 (31, 32). To further clarify whether the Akt/mTOR pathway was involved in mediating the effect of KM, the expression levels of p-Akt, Akt, p-mTOR, mTOR were assessed. With the inhibition of HK2 expression, the p-mTOR and p-Akt also reduced that indicated Akt/mTOR pathway could be of great importance during the downregulation of HK2.

Although herbal medicine has provided enormous potential lead compound to be applied to healthcare because of their structural diversity, complex scaffolds of compounds, low solubility, metabolic instability and ambiguous mechanisms of action has impeded them to achieve the final candidate drug (33, 34). It is interesting to optimize the structure of KM to increase the binding ability to receptor, raise the activity strength and selectivity. Vinyl is present in the active site which is a large space and mainly composed of hydrophobic amino acid. It is quite possible that substitution vinyl with favorable lipophilic groups, such as phenyl or benzoyl group is beneficial to better affinity as well as greater activity. Furthermore, it is also reasonable to design KM analogue by replacing the tricyclo fragment linking a vinyl group with lipophilic side chain to simplify the complicated ring system. Such further structure development and optimization of KM may contribute to the superior anti-CRC potency.

## Conclusion

Effective ingredient and corresponding target validation are the key steps in herbal drug development chain. In this study, it adopts synthetically network pharmacology, molecular docking, transcriptomics and bioinformatics approach for mapping the anti-CRC ingredient and mechanism of *G. elegans*. It gives a novel and efficient strategy to explore the pharmacological mechanism of herbal medicine. As a result, we found that KM had a potent anti-CRC capacity through regulating glycolysis and VDAC-1 association *via* targeting PDK1. AKT/mTOR/HK2 signaling down-regulation may play an essential role in this process. Replacement of vinyl group with lipophilic group will provide rational direction for structure optimization study in the future.

## Data Availability Statement

The datasets presented in this study can be found in online repositories. The names of the repository/repositories and accession number(s) can be found below: NCBI, PRJNA670048.

## Ethics Statement

The animal study was reviewed and approved by China Medical University Institutional Animal Care and Use Committee.

## Author Contributions

LW conducted the experiments and participated in the conception and the design of the study. HX and YD conducted the experiments and performed the analysis. LW and JL contributed to analyze the data and draft the manuscript. FM contributed to the conception, design, and analysis of the manuscript. All authors contributed to the article and approved the submitted version.

## Funding

This work was supported by the National Nature Science Foundation of China (81703405, 81573687). Key R&D Projects in Liaoning Province (2019026).

## Conflict of Interest

The authors declare that the research was conducted in the absence of any commercial or financial relationships that could be construed as a potential conflict of interest
