# Supplementary material for: An Integrated Network, RNA Sequencing, and Experiment Pharmacology Approach Reveals the Active Component, Potential Target, and Mechanism of Gelsemium elegans in the Treatment of Colorectal Cancer
Source: Front Oncol. 2020 Dec 23;10:616628. doi: 10.3389/fonc.2020.616628 (PMC7786369; doi:10.3389/fonc.2020.616628)
Supplement: Supplementary file 1 [file DataSheet_1.zip › supplementary material 2/supplementary.docx]

Table 1. GO enrichment analysis for targets of *G.elegans.*

Table 2. KEGG enrichment analysis for targets of *G.elegans*.

Table 3. The dock results of 7 compounds to the protein PDK1 and TP53

| Compound | Target | Score |
| --- | --- | --- |
| Koumine | PDK1 | -9.1004 |
| 21-Oxogelsemine | TP53 | -8.4248 |
| Gelsedine | PDK1 | -8.0302 |
| Gelsevirine | PDK1 | -7.4206 |
| Koumine | TP53 | -7.3187 |
| Gelsenicine | TP53 | -7.0256 |
| Gelsemine | PDK1 | -6.8734 |
| Gelsemine | TP53 | -6.5039 |
| Gelsemicine | TP53 | -6.4413 |
| Gelsevirine | TP53 | -5.8712 |
| Gelsedine | TP53 | -5.7213 |
| 21-Oxogelsemine | PDK1 | -5.4126 |
| Gelsemicine | PDK1 | -5.2412 |
| Gelsenicine | PDK1 | -5.1109 |

Table 4. downregulated differential expression genes after KM treatment

Table 5. upregulated differential expression genes after KM treatment

**
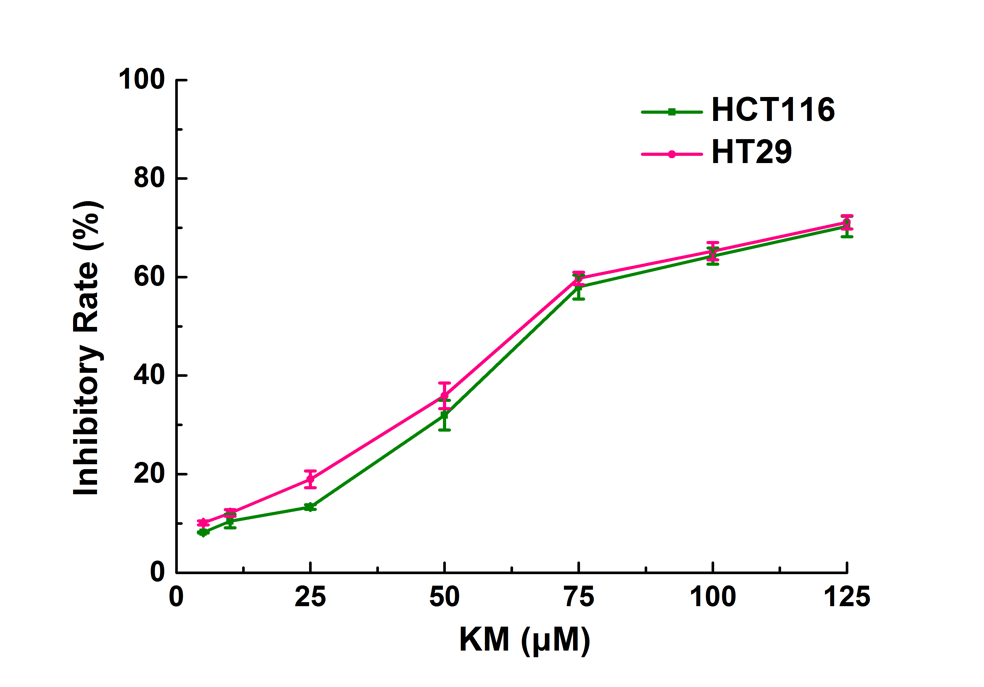
**

**FIGURE.1** KM inhibits cell growth in a panel of human colorectal cells, including HCT116 and HT29

**FIGURE 1**: GO and KEGG signaling pathway enrichment of screened genes. “Rich factor” represents the ratio of the number of target genes belonging to a pathway and the number of the annotated genes located in the pathway. The size of the dot indicates the number of target genes in the pathway and the color of the dot reflects the different p values.

**Information of the primary antibodies**

Bcl-2: (Abbkine,California,USA)

Bax : (Abbkine,California,USA)

Cytc: (ABclonal Technology,China)

β-actin:(Proteintech Group, Chicago,USA）

Akt: (Wanleibio, China)

P-Akt:(Wanleibio, China)

mTOR:(Cell signaling technology, Boston,USA)

P-mTOR: (Cell signaling technology, Boston,USA)

HK2: (Abcam,England)

PDK1:(Santa Cruz Biotechnology, CA, USA)

**Reverse transcription-quantitative polymerase chain reaction (RT-*q*PCR)**

Isolated RNA was reverse transcribed into cDNA using by the ReverTra Ace *q*PCR RT kit (TOYOBO CO., LTD.) according to the manufacture’s manual. The cDNA samples were amplified and quantified using SYBR ^®^ Green Realtime PCR Master Mix kit (Takara Bio, Shiga, Japan). Corresponding primers sequences were listed in Table 6. The comparative delta-delta CT (2^-ΔΔ^*^Ct^*) method was used to calculate the relative expression levels with GAPDH as internal controls for normalization.

Table 6. Corresponding primers sequences for RT-qPCR

| ANGPT2 | F-CAACACTCAGTGGCTAATGAAG |
| --- | --- |
|  | R-GCATTCTGCTGTATCTCTACCA |
| NTRK1 | F-ATCTACAGCACCGACTATTACC |
|  | R-GGTGAACTTACGGTACAGGATG |
| SLC2A3 | F-TTCAATGCTGATTGTCAACCTG |
|  | R-GCATTTCAACCGACTTAGCTAC |
| EDN1 | F-TAGCCAAAAAGACAAGAAGTGC |
|  | R-TTCTTCCTCTCACTAACTGCTG |
| HMGCS1 | F-AAACTCTCCATACAGTGCTACC |
|  | R-GTGAGTGAAAGATCATGAAGCC |
| HK2 | F-CGACAGCATCATTGTTAAGGAG |
|  | R-GCAGGAAAGACACATCACATTT |


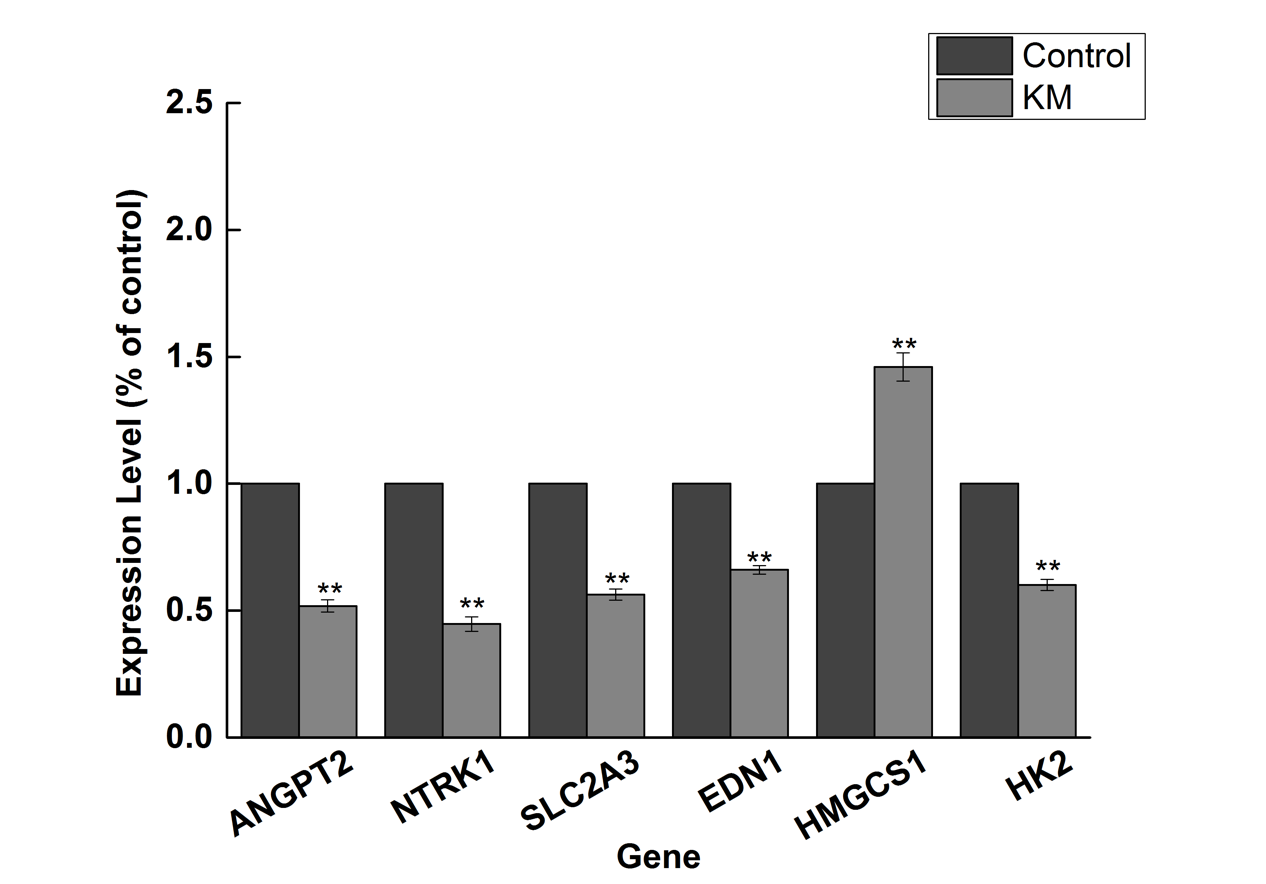


FIGURE 2. Analysis of the expression level of representative DEGs using RT-qPCR. Each value is the mean ± SD of three separate experiments. The statistically significant differences are indicated with ***p* < 0.01 compared with the control grou
